# Acute liver injury is triggered by dysbiosis and microbial translocation involving gut–liver axis

**DOI:** 10.1080/19490976.2026.2749977

**Published:** 2026-10-19

**Authors:** Urmi Halder, Hamida Hamida, Khadija Kakar, Xiaoming Yang, Yin Zhong, Mitzi Nagarkatti, Prakash Nagarkatti

**Affiliations:** a Department of Pathology, Microbiology and Immunology, University of South Carolina School of Medicine, Columbia, SC, USA

**Keywords:** Bacteriome, hepatotoxins, virome, systemic circulation, microbiota translocation, inflammatory response

## Abstract

Acute liver injury (ALI) is a severe clinical condition that can progress to chronic liver failure, cirrhosis, or hepatocellular carcinoma, yet therapeutic options remain limited. Here, we investigated how hepatotoxin-induced gut–liver dysbiosis drives hepatic inflammation through microbial crosstalk. Using three murine ALI models, we assessed liver injury, intestinal barrier integrity, microbial composition along the gut–liver axis, and immune activation. Each hepatotoxin recapitulated distinct features of human ALI and induced unique patterns of hepatic and intestinal injury, enabling the progressive translocation of live bacteria from the gut to the liver and bloodstream. ALI was consistently associated with depletion of commensal taxa (*Lactobacillaceae*, *Ruminococcaceae*, and *Bifidobacteriaceae*) and enrichment of opportunistic pathogens (*Enterococcaceae*, *Clostridiaceae*, *Enterobacteriaceae*, and *Burkholderiaceae*). Dysbiosis also involved reduced gut bacteriophages and the expansion of viral families, including *Retroviridae* and *Herpesviridae*, in the liver and blood. Transplantation of the fecal or liver microbiota from ALI mice into microbiota-depleted recipients reproduced liver injury, immune activation, and intestinal barrier disruption, demonstrating that dysbiotic microbial communities alone are sufficient to trigger hepatic inflammation. These communities activated Th_1_/Th_17_ associated pathways (*NF-κB*, *IL-17*, *TGF-β*, and antigen presentation networks) and expanded pro-inflammatory immune populations. A key advance of this study is the direct comparison of fecal- versus liver-derived microbiota, revealing distinct mechanistic roles for each in driving liver pathology. Across all models, hepatotoxin-induced intestinal barrier breakdown drives gut–liver dysbiosis, microbial translocation, and immune-mediated liver injury. These findings uncover a unifying mechanism by which microbial imbalance reprograms hepatic immunity and accelerates ALI progression.

## Graphical abstract

**Figure:**
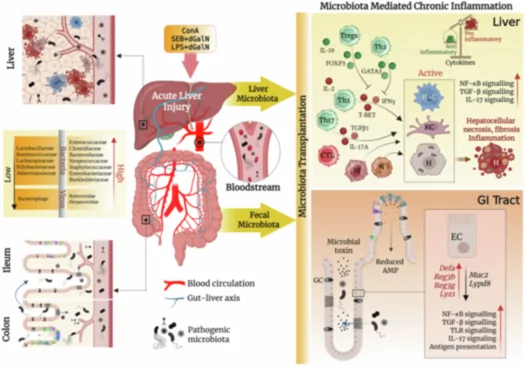

Hepatotoxin-induced dysbiosis disrupts the gut barrier, enabling microbial translocation to the liver via the gut–liver axis and bloodstream. Tregs: regulatory T cell, Th_1_: T helper 1, Th_2_: T helper 2, Th_17_: T helper 17, CTL: cytotoxic T lymphocyte, M: macrophage, N: neutrophil, H: hepatocyte, KC: Kupffer cell, HSC: hepatic stellate cell, EC: epithelial cell, GC: goblet cell, AMP: antimicrobial peptide. (Created in https://BioRender.com).

## Introduction

Acute liver injury (ALI) is a life-threatening condition characterized by rapid hepatocellular damage and loss of liver function.^1-4^ Some examples of ALI include drug-induced liver injury, in which an overdose of compounds such as acetaminophen (APAP) or idiosyncratic reactions to other medications cause liver failure.^5^ ^,^ ^6^ Additionally, viral infections such as Hepatitis A, B, and E can trigger acute liver failure.^7-10^ Consumption of poisonous mushrooms such as *Amanita phalloides* can also cause ALI and failure, primarily due to a group of toxins called amatoxins.^11^

While liver injury can result directly from hepatotoxins, the gut microbiota and dysbiosis can be a contributing factor to ALI, particularly in cases triggered by sepsis.^12-14^ The gut microbiome plays a critical role in maintaining human health, and growing evidence links gut dysbiosis to a wide spectrum of hepatic disorders. A compromised intestinal barrier allows pathogenic microbes and their metabolites to translocate to the liver and blood circulation, contributing to a wide range of chronic liver diseases.^15-19^ Sepsis and other illnesses can cause a “leaky gut,” where the intestinal barrier becomes more permeable. This allows bacteria and their inflammatory byproducts, such as LPS or endotoxins, to pass from the gut into the bloodstream and liver, triggering a cascade of inflammation, cell death, and fibrosis.^20-27^ In addition to the bacteria, some viruses found in the gut can also contribute to ALI through the gut‒liver axis, especially when the intestinal barrier is compromised.^28-30^

Recent research has suggested that a distinct, low-biomass microbiota also resides in the liver of normal individuals.^31^ This discovery highlights the intricate, bidirectional relationship of the gut‒liver axis, which is active even under healthy conditions. The liver microbiota is selectively populated from the gut through the portal vein, which carries venous blood directly from the intestines to the liver.^32^

Many toxins and their metabolites cause liver injury by directly damaging hepatocytes.^33^ ^,^ ^34^ For example, many drugs and chemicals are metabolized by the liver's cytochrome *P450* enzyme system. This process can produce highly reactive and toxic intermediates that can cause ALI.^35^ ^,^ ^36^ On the other hand, many hepatotoxins also damage the intestinal barrier, allowing bacteria and their products to enter the liver and trigger an inflammatory response. To investigate the contribution of gut–liver microbial interactions to ALI, we employed three complementary murine models that induce liver injury through distinct mechanisms: concanavalin A (ConA), which models T cell-mediated autoimmune hepatitis;^37-40^ staphylococcal enterotoxin B (SEB) combined with D-galactosamine (dGalN), which induces superantigen-driven cytokine storm and hepatocyte sensitization;^41-44^ and LPS combined with dGalN, which activates Toll-like receptor 4-mediated innate immune responses.^45-48^ Although these models reproduce key pathological features of human ALI, the contribution of gut- and liver-associated bacterial and viral communities to hepatotoxin-induced liver injury remains poorly understood.

In the current study, we investigated whether hepatotoxin-induced ALI alters the microbiota of the gut, blood, and liver, resulting in microbial translocation, intestinal and hepatic pathology, and heightened immune response. Using fecal and liver microbiota transplantation, transcriptomic profiling, and immune phenotyping, we further evaluated how acute microbiota dysbiosis contributes to hepatic remodeling and systemic immune activation. Our results reveal a shared pathway by which microbial dysbiosis and translocation alters hepatic immunity, thereby driving the progression of acute liver injury.

## Results

### Hepatotoxins significantly induces acute liver injury (ALI)

In the current study, we compared 3 models of ALI triggered by hepatotoxins, such as ConA, SEB+dGalN, or LPS+dGalN. To determine the extent of liver injury, both serum alanine aminotransferase (ALT) and aspartate aminotransferase activity (AST) were measured at 24 h and 48 h after toxin challenge and compared with those of vehicle controls. Distinct hepatotoxins induced variable levels of ALI and AST. ConA administration led to a significant elevation in the serum ALT and AST at 24  h, higher than those in SEB+dGalN- or LPS+dGalN-treated groups; however, both ALT and AST levels declined by 48  h. In SEB+dGalN-treated mice, ALT and AST levels remained moderately elevated but stable over time. In contrast, LPS+dGalN challenged mice exhibited a progressive increase in both ALT and AST, with higher values observed at 48 h compared with 24 h (Figure 1a).

**Figure 1.**
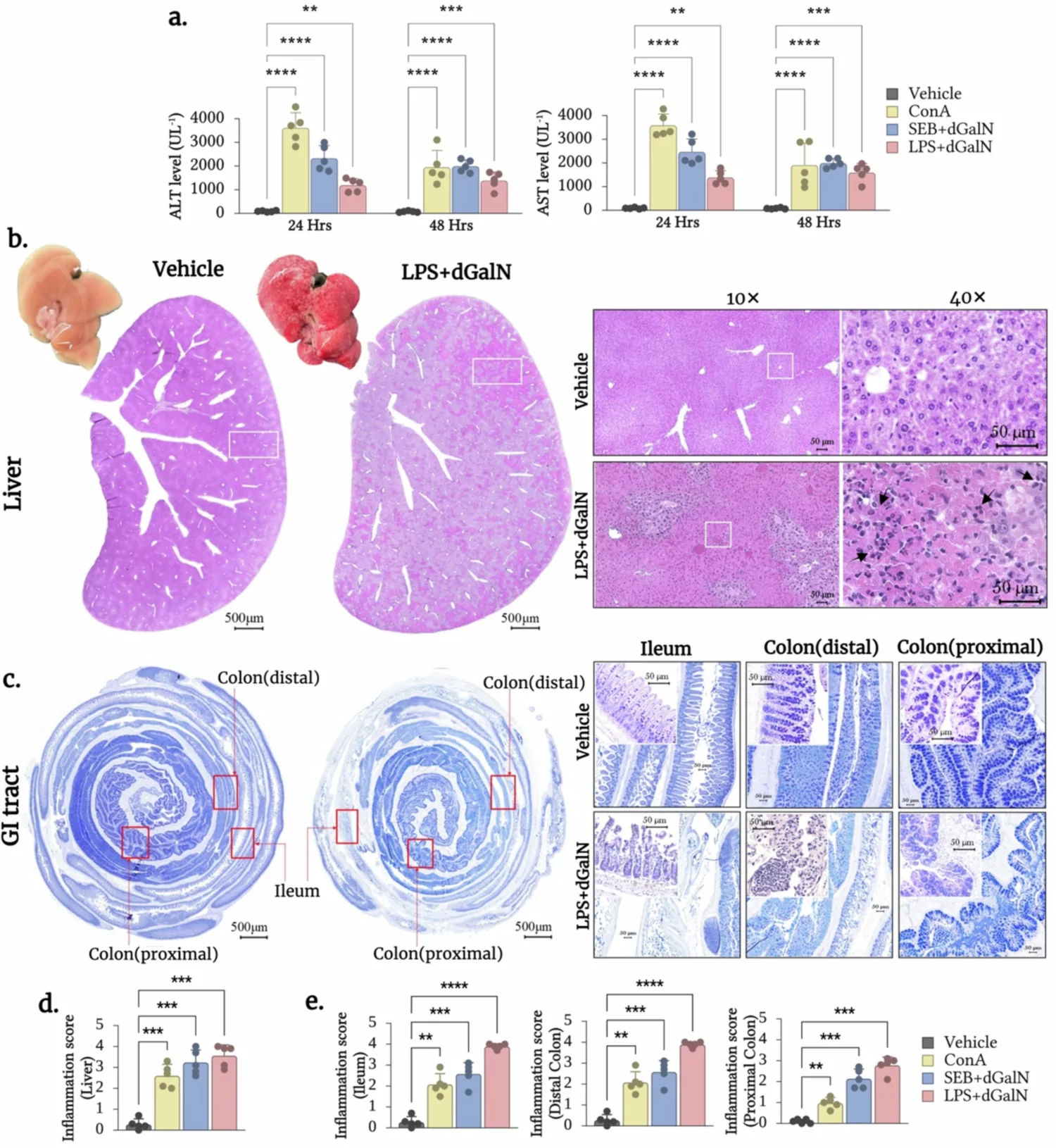
Hepatotoxin-induced ALI and gut inflammation. The mice were injected with Vehicle, ConA, SEB+dGalN, or LPS+dGalN as described in the Methods. At 24 and 48 h time points, the mice were examined for liver and intestinal injury. (a) Serum ALT and AST levels. (b) Representative gross liver morphology and H&E-stained large lobe section overview of livers at 10× magnifications and a detailed histopathology of those liver sections at 10× and 40× magnifications. (c) Representative cross-section overview (10× magnification) of the Swiss rolled GI tracts stained with PAS-AB and H&E at 10× and 20× magnifications shows the regions of ileum, proximal colon, and distal colon. Quantitative histopathological ranking of (d) liver and (e) GI tract at 10× and 40× magnification according to the criteria listed in Supplemental Table 1. Statistical significance was analyzed using a one-way ANOVA or Student’s t-test. *p < 0.05, **p < 0.01, ***p < 0.001, and ****p < 0.0001.

Histopathological evaluation further supported the extent of liver injury. Gross examination and hematoxylin and eosin (H&E)-stained liver sections showed that the LPS+dGalN group exhibited the most extensive hepatic injury, characterized by widespread hepatocyte apoptosis/necrosis, hemorrhage, and disruption of lobular architecture. In comparison, the ConA and SEB+dGalN groups displayed focal hepatocellular necrosis with moderate inflammatory cell infiltration (Figure 1b; Supplemental Figure 2). Qualitative histopathological assessment of H&E-stained liver sections further demonstrated normal hepatic architecture with minimal inflammatory infiltrates in the Vehicle group. In contrast, all treatment groups exhibited increased inflammatory cell infiltration and hepatocellular injury relative to the Vehicle group, with inflammatory infiltrates appearing more pronounced in the SEB+dGalN group than in the ConA group and comparable to those observed in the LPS+dGalN group (Figure 1d).

Examination of the gastrointestinal (GI) tract demonstrated parallel injury in the small and large intestines. Periodic acid–Schiff/Alcian blue (PAS-AB) staining of ileal and colonic tissues revealed intact mucin layers and abundant goblet cells in the Vehicle group, whereas ConA, SEB+dGalN, and LPS+dGalN induced varying degrees of epithelial disruption and goblet cell depletion. Histopathological changes were most pronounced in the ConA group, followed by the SEB+dGalN and LPS+dGalN groups, as evidenced by villus blunting, crypt distortion, disrupted mucin integrity, and reduced goblet cell numbers in the ileum and both proximal and distal colon (Figure 1c; Supplemental Figure 3). Qualitative histopathological assessment further demonstrated increased inflammatory cell infiltration, epithelial injury, and disruption of mucosal architecture in all treatment groups compared with the Vehicle group. These inflammatory changes were most severe in the ConA group, followed by the SEB+dGalN and LPS+dGalN groups, whereas the Vehicle group maintained normal intestinal morphology with preserved villus and crypt architecture (Figure 1e). Overall, these results showed that all three hepatotoxins induce liver and intestinal injury, but the magnitude and persistence of damage were greatest with LPS+dGalN, which produced progressive hepatocellular necrosis alongside pronounced disruption of intestinal barrier function.

### ALI induced dysbiosis of bacteria

The fact that liver injury in all 3 models was associated with significant intestinal pathology, we next investigated dysbiosis in the gut, liver, and blood. To that end, we cultured bacterial species from harvested liver tissue, blood, and gut (intestine) content at 24 and 48  h post-toxin challenge under both aerobic and anaerobic conditions. Vehicle-treated mice showed negligible bacterial growth differences at 24 and 48  h, whereas ConA, SEB+dGalN, and LPS+dGalN challenged mice exhibited significantly increased colony-forming units (CFUs) in liver, blood, and gut samples. Notably, the bacterial load was higher in LPS+dGalN mice compared with ConA and SEB+dGalN groups. Furthermore, live bacterial counts in both liver and blood were markedly elevated at 48 h relative to 24 h, indicating progressive microbial translocation over time (Supplemental Figure 1). These findings suggested the spreading of bacterial species from the gut through the gut–liver axis or portal circulation to systemic sites as a consequence of intestinal barrier loss. To further characterize these microbial changes, shotgun metagenomic sequencing was performed after 48 h of disease induction to analyze the liver, blood, and gut microbiome.

Liver, blood, and gut exhibited distinct bacterial communities during ALI. *α*-diversity analysis revealed that bacterial richness was significantly increased in the liver and blood after ConA, SEB+dGalN, or LPS+dGalN challenge compared with vehicle controls, while species evenness in the gut decreased with disease progression as measured by the Shannon and Simpson indices (Figure 2a). *β*-diversity using Bray–Curtis dissimilarity indices showed distinct clustering of microbial communities in the liver, blood, and gut of ALI mice compared with vehicle control, indicating compartment-specific shifts (Figure 2b).

**Figure 2.**
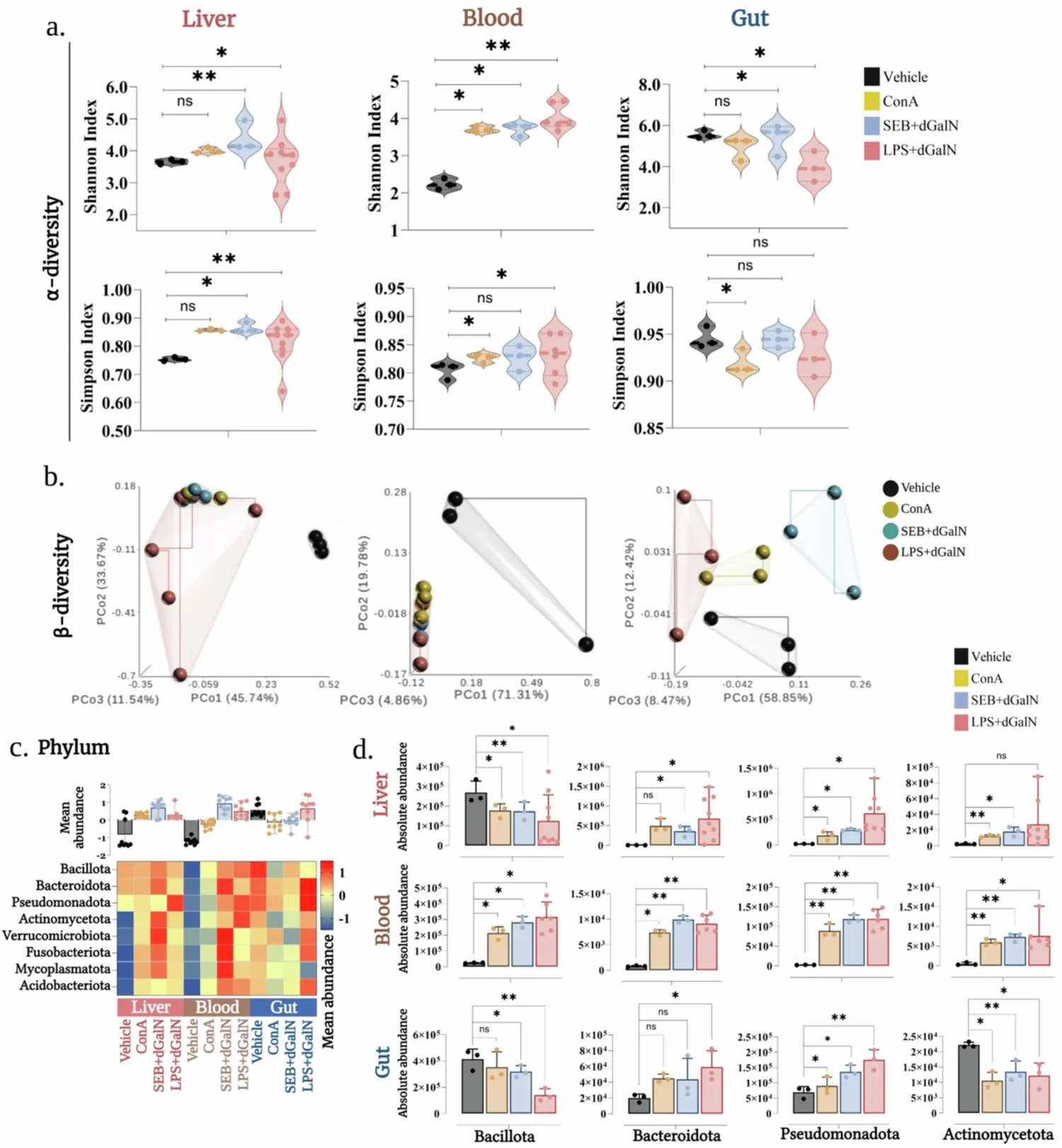
Distinct bacterial communities populated in the liver, blood, and gut during ALI. The mice were injected with Vehicle, ConA, SEB+d-GalN, or LPS+d-GalN as described in the Methods. Microbial diversity and composition in the liver, blood, and gut were analyzed using metagenome sequencing. (a) *α*-diversity (Shannon and Simpson indices). (b) *β*-diversity (Bray–Curtis dissimilarity indices). (c) Mean abundance of bacterial taxa at the phylum level (Z-score; *p* ≤ 0.05). (d) Absolute abundance of phyla: *Bacillota*, *Bacteroidota*, *Pseudomonadota*, and *Actinomycetota*. Statistical significance was analyzed using a one-way ANOVA or Wilcoxon rank-sum test. *p < 0.05, **p < 0.01, ***p < 0.001, and ns = non-significant.

After toxin challenge, the mean bacterial abundance increased by ~2-fold in the liver and blood but decreased in the gut. In vehicle-treated mice, *Bacillota, Bacteroidota*, and *Pseudomonadota* dominated the gut, while the liver was enriched with *Bacteroidota* and *Bacillota*, and the blood was dominated by *Bacillota* and *Bacteroidota*. In ConA, SEB+dGalN, and LPS+dGalN challenged mice, the relative abundance of *Pseudomonadota* and *Bacteroidota* increased in the liver, blood, and gut, accompanied by a significant reduction of *Bacillota* and *Actinomycetota* in the gut compared with vehicle controls (Figure 2c,d). In the liver, the mean abundance of the bacterial orders *Clostridiales, Bacillales, Bacteroidales, Flavobacteriales, Pseudomonadales, Alteromonadales, Enterobacterales, Burkholderiales,* and *Xanthomonadales* increased by approximately 3-fold in LPS+dGalN-treated mice, whereas a ~1.5-fold increase was observed in the ConA and SEB+dGalN-treated groups compared with the vehicle controls. In the blood, the abundance of *Xanthomonadales, Enterobacterales, Bacillales, Clostridiales, Flavobacteriales, Cytophagales*, and *Verrucomicrobiales* was consistently higher in mice with ALI. By contrast, in the gut, the mean abundance of dominant orders such as *Lactobacillales, Clostridiales, Flavobacteriales, Alteromonadales, Streptomycetales, Bifidobacteriales, Verrucomicrobiales*, and *Eubacteriales* was significantly decreased in ConA, SEB+dGalN, and LPS+dGalN challenged mice compared with vehicle (Figure 3a). At the family level, vehicle-treated mice showed enrichment of *Lactobacillaceae, Ruminococcaceae, Lachnospiraceae, Bifidobacteriaceae,* and *Akkermansiaceae*. These families were markedly depleted in the gut following toxin challenge, whereas *Enterococcaceae, Clostridiaceae, Bacteroidaceae, Streptococcaceae, Staphylococcaceae, Enterobacteriaceae,* and *Burkholderiaceae* were significantly increased in both liver and blood of ALI mice (Figure 3b). In vehicle-treated mice, gut microbial communities were dominated by genera such as *Eubacterium, Lachnoclostridium, Butyrivibrio, Alistipes, Exiguobacterium, Faecalibacterium, Akkermansia, Roseburia,* and *Lactobacillus*. These genera were markedly reduced in toxin-challenged mice. In contrast, genera including *Enterococcus*, *Xanthomonas*, *Staphylococcus*, *Klebsiella*, *Burkholderia*, *Salmonella*, *Serratia, Clostridium*, *Pseudomonas*, and *Acinetobacter* were significantly enriched in both liver and blood of ConA, SEB+dGalN, and LPS+dGalN-treated mice (Figure 3c).

**Figure 3.**
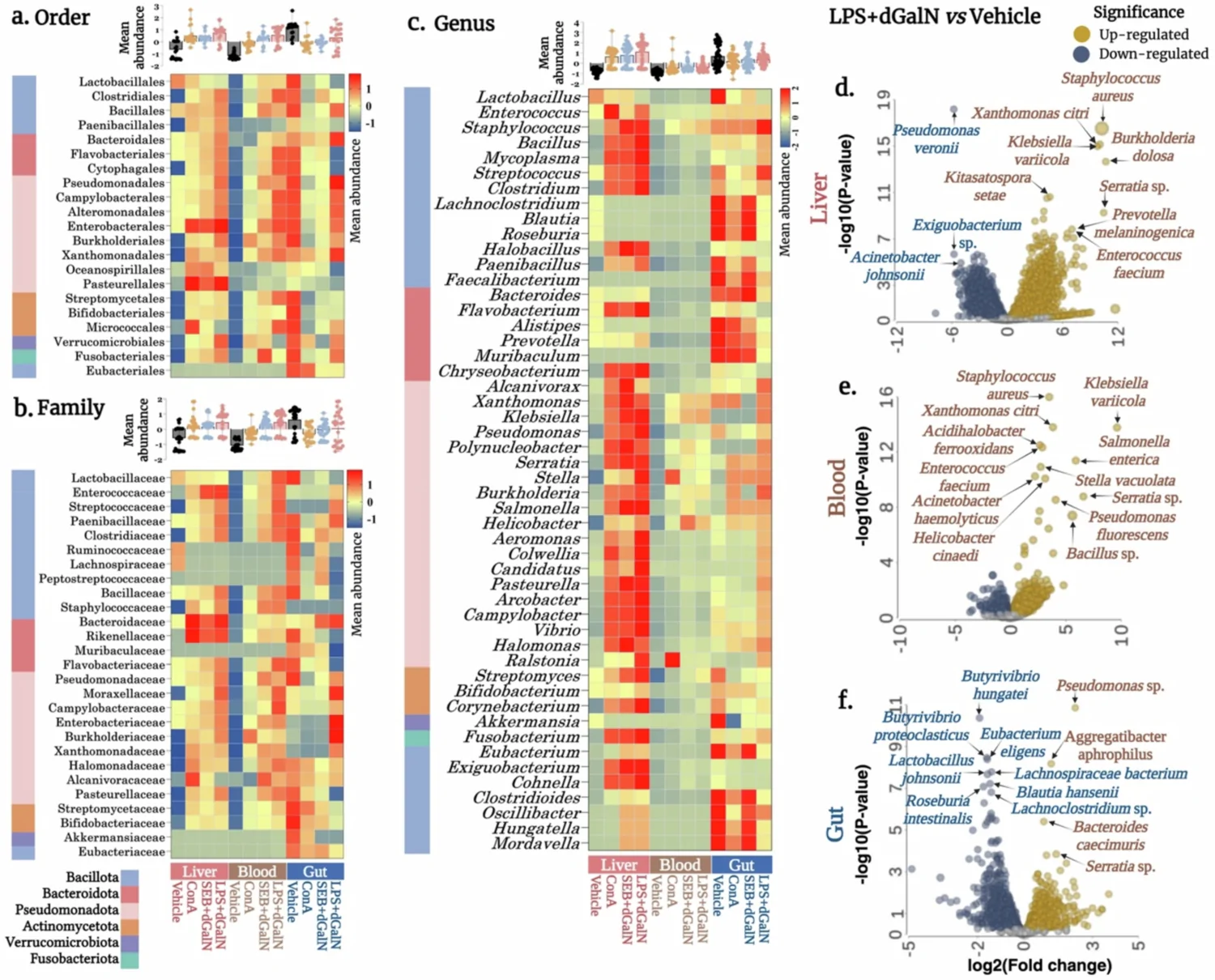
Taxonomic shifts and differential abundance of microbial communities in the liver, blood, and gut. The mice were injected with Vehicle, ConA, SEB+d-GalN, or LPS+d-GalN as described in the Methods. Microbial diversity and composition in the liver, blood, and gut were analyzed using metagenome sequencing. (a–c) Bacterial mean abundance (Z-score, *p* ≤ 0.05) at the (a) order, (b) family, and (c) genus levels. (d–f) Representative volcano plots of differentially abundant bacterial species in diseased mice versus vehicle controls (fold change ≥ 1; *p* ≤ 0.05).

At the species level, in contrast to vehicle control, ConA, SEB+dGalN, and LPS+dGalN treated mice showed upregulation of *Staphylococcus aureus*, *Xanthomonas citri*, *Klebsiella variicola*, *Burkholderia dolosa*, *Enterococcus faecium*, *Serratia* sp., *Kitasatospora setae*, and *Prevotella melaninogenica*, while commensal species such as *Pseudomonas veronii*, *Exiguobacterium* sp., and *Acinetobacter johnsonii* were downregulated in the liver (Figure 3d; Supplemental Figure 4a). In blood, *Staphylococcus aureus*, *Klebsiella variicola*, *Xanthomonas citri*, *Enterococcus faecium*, *Salmonella enterica*, *Acidihalobacter ferrooxidans*, *Acinetobacter haemolyticus*, *Pseudomonas fluorescens*, *Helicobacter cinaedi*, *Bacillus* sp., and *Serratia* sp. were significantly upregulated in ALI mice as compared to vehicle (Figure 3e; Supplemental Figure 4b). In the gut, commensals such as *Butyrivibrio hungatei*, *Butyrivibrio proteoclasticus*, *Eubacterium eligens*, *Lactobacillus johnsonii*, *Roseburia intestinalis*, *Blautia hansenii*, *Lachnoclostridium* sp., and species of *Lachnospiraceae* were significantly reduced, while opportunistic taxa including *Pseudomonas* sp., *Bacteroides caecimuris*, and *Serratia* sp. were enriched in all ALI mice as compared to vehicle (Figure 3f; Supplemental Figure 4c).

Notably, across taxonomic levels from phylum to species, ConA and SEB+dGalN induced microbial alterations resembled those observed with LPS+dGalN but with lower magnitude. Together, these data demonstrated that hepatotoxin-induced liver injury is characterized by a loss of gut commensals and enrichment of opportunistic pathogens in the liver and blood, consistent with microbial translocation and dysbiosis-driven systemic dissemination.

### ALI induced dysbiosis of viruses

Distinct communities of viruses were also observed in the liver, blood, and gut during ALI. The *α*-diversity of viruses in the liver and blood of ConA, SEB+dGalN, and LPS+dGalN challenged mice showed an increase in richness and reduced evenness, whereas in the gut, both richness and evenness were reduced compared to vehicle controls (Figure 4a). The *β*-diversity analysis revealed distinct clustering of viral communities in the liver, blood, and gut of toxin-challenged groups compared with vehicle, paralleling the bacterial community shifts observed earlier (Figure 4b). Viral species richness was highest in the liver and blood of LPS+dGalN treated mice, while the gut exhibited a significant loss of richness in this group relative to ConA or SEB+dGalN.

**Figure 4.**
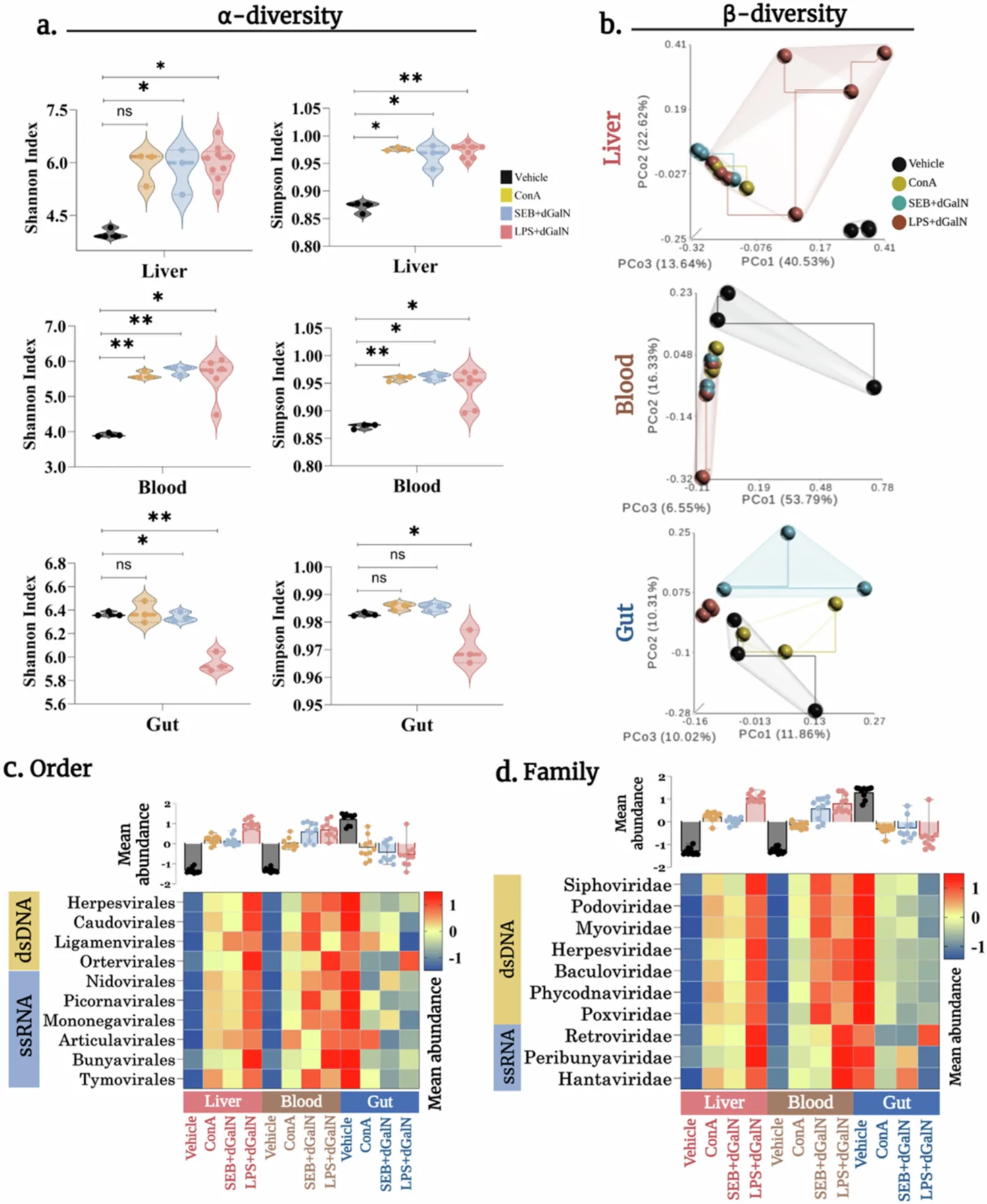
Viral diversity and taxonomic dysregulation across the liver, blood, and gut during ALI. The mice were injected with Vehicle, ConA, SEB+d-GalN, or LPS+d-GalN as described in the Methods. The viral communities in the liver, blood and gut were analyzed by metagenomic sequencing. (a) *α*-diversity (Shannon and Simpson indices). (b) *β*-diversity (Bray–Curtis dissimilarity). (c) Order-level mean abundance (Z-score, *p* ≤ 0.05) and (d) Family-level mean abundance (Z-score, *p* ≤ 0.05). Statistical significance was analyzed using a one-way ANOVA or Wilcoxon rank-sum test. *p < 0.05, **p < 0.01, ***p < 0.001, and ns = non-significant.

At the order level, total viral mean abundance increased in the liver of ConA, SEB+dGalN, and LPS+dGalN-treated mice compared with vehicle, with ~2-fold enrichment from baseline in the LPS+dGalN group. *Caudovirales, Ortervirales, Herpesvirales, Bunyavirales, Muvirales, Mononegavirales,* and *Goujianvirales* were among the most abundant orders in the liver, followed by SEB+dGalN and ConA groups (Figure 4c). In the blood, *Herpesvirales, Ortervirales*, and *Bunyavirales* were highly abundant, while overall viral abundance was reduced in the gut of diseased mice. The absolute abundance of *Ortervirales, Herpesvirales,* and *Caudovirales* was significantly increased in both liver and blood of ALI mice but decreased in gut, with the strongest effect in LPS+dGalN treated animals (Supplemental Figure 5a). At the family level, total viral abundance was significantly higher in liver of LPS+dGalN mice followed by SEB+dGalN and ConA. Dominant families included *Hantaviridae, Retroviridae, Peribunyaviridae, Siphoviridae, Herpesviridae, Flaviviridae, Baculoviridae, Nudiviridae, Adenoviridae, Rhabdoviridae, Poxviridae, Phycodnaviridae, Papillomaviridae, Podoviridae, Myoviridae,* and *Coronaviridae* (Figure 4e). In blood, *Hantaviridae, Retroviridae, Peribunyaviridae,* and *Alloherpesviridae* were dominant. The absolute abundance of *Podoviridae, Retroviridae, Poxviridae, Myoviridae*, and *Siphoviridae* was significantly increased in the liver and blood of ALI mice, but these families were reduced in the gut (Supplemental Figure 5b). In vehicle controls, viral species were relatively balanced across compartments, with moderate representation of ssRNA viruses, including *Orthobornavirus, Betacoronavirus,* and *Orthohantavirus*, and dsDNA viruses, such as *Polyomavirus, Papillomavirus*, and *Adenovirus*. Following hepatotoxin challenge, particularly in LPS+dGalN treated mice, there was a marked increase in dsDNA viral species in the liver, including *Mastadenovirus, Herpesvirus, Iridovirus, Polyomavirus*, and *Podovirus*. Similarly, ssRNA viruses such as *Betacoronavirus, Orthohantavirus*, and *Flavivirus* were enriched in the liver and blood of ALI mice. In contrast, the gut samples showed an overall reduction in both ssRNA and dsDNA viral species compared with vehicle, indicating a loss of viral richness in intestinal communities (Supplemental Figure 5c). These patterns suggest that acute liver injury drives the expansion of systemic viral species while reducing viral abundance in the gut, paralleling bacterial dysbiosis and supporting the role of virome alterations in gut–liver axis dysfunction.

### Functional profiling of microbial communities during ALI

Functional metagenomic profiling revealed significant differences in microbial pathways across the vehicle control, ConA, SEB+dGalN, and LPS+dGalN-treated groups. In terms of metabolism, hepatotoxin-challenged mice showed enrichment of pathways related to carbohydrate metabolism, amino acid metabolism, cofactors and vitamins, lipid metabolism, glycan biosynthesis, xenobiotic degradation, and nucleotide metabolism compared with vehicle controls. The enrichment was most pronounced in LPS+dGalN- treated mice, followed by SEB+dGalN and ConA groups (Supplemental​​​​​ Figure 6a). In addition, disease-associated pathways were highly enriched in toxin-challenged mice, including bacterial invasion of epithelial cells, *Staphylococcus aureus* infection, *Herpes simplex virus 1* infection, *Hepatitis C*, and epithelial cell signaling in *Helicobacter pylori* infection. Multiple antimicrobial resistance pathways, such as *β*-lactam, vancomycin, and cationic antimicrobial peptide (CAMP) resistance, were also detected, with the highest abundance in LPS+dGalN mice (Supplemental​​​​​​ Figure 6b). Taken together, LPS+dGalN induced acute liver injury caused more pronounced alterations in gut and liver microbial communities compared with ConA or SEB+dGalN induced injury.

### Microbiota transplantation

To test if the dysbiosis seen in mice with ALI was instrumental in inducing inflammation and, in turn, causing liver injury, we performed microbiota transplantation experiments in the LPS model (Figure 5a). Briefly, mice were treated with an antibiotic cocktail for 4 weeks to deplete the microbiota, which was confirmed by a significant reduction in CFU counts in both the liver and gut compared with baseline (Figure 5b). Subsequently, microbiota transplantation was performed, and successful repopulation was verified by increased CFU counts in the liver and gut 7 d post-transplantation (Figure 5c). We tested 3 groups of mice for the ability of transplanted microbiota to induce liver damage in microbiota-depleted mice. (1) Vehicle _(fecal)_: microbiota-depleted mice that received fecal microbiota from vehicle control mice, (2) LPS _(liver)_: microbiota-depleted mice that received liver microbiota from LPS-induced ALI mice. (3) LPS _(fecal)_: microbiota-depleted mice that received fecal microbiota from LPS-induced ALI mice.

**Figure 5.**
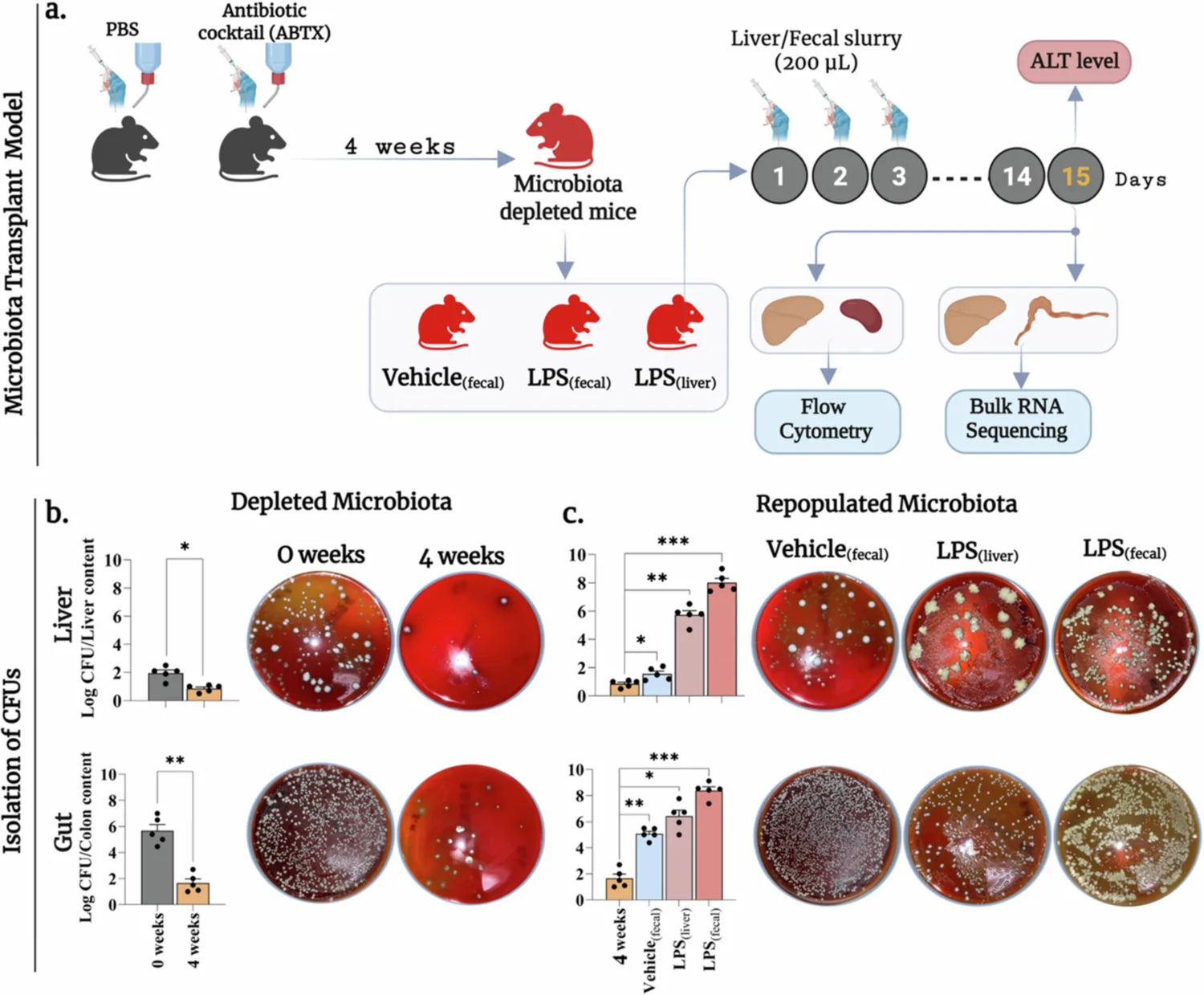
Microbiota transplantation model to assess gut–liver microbial interactions during ALI. Mice were treated with an antibiotic cocktail to deplete the microbiota, followed by transplantation with Vehicle _(fecal)_ (fecal slurry from vehicle-treated mice), LPS _(fecal)_ (fecal slurry from LPS+d-GalN-challenged mice), or LPS _(liver)_ (liver slurry from LPS+d-GalN-challenged mice). CFUs from the gut and liver content were analyzed. (a) Schematic overview of the microbiota transplantation workflow. (b) CFU counts and representative plates (10^−1^ dilution) after microbiota depletion. (c) CFU counts and representative plates (10^−1^ dilution) after microbiota transplantation. Statistical significance was analyzed using a one-way ANOVA or Student’s t-test. *p < 0.05, **p < 0.01, and ***p < 0.001.

### Fecal- and liver-derived microbiota promote hepatic inflammation

Interestingly, the serum ALT and AST levels were significantly elevated in both LPS _(liver)_ and LPS _(fecal)_ groups when compared to Vehicle _(fecal)_ group (Figure 6a,b). Histopathological analysis of the liver revealed normal hepatic architecture in Vehicle _(fecal)_ mice, whereas LPS _(liver)_ group showed hepatocyte swelling, necrosis, and mild collagen deposition. Moreover, the fecal-derived microbiota caused extensive hepatocyte necrosis, pronounced inflammatory infiltration, and marked fibrosis, as seen in LPS _(fecal)_ group (Figure 6b; Supplemental Figure 7a).

**Figure 6.**
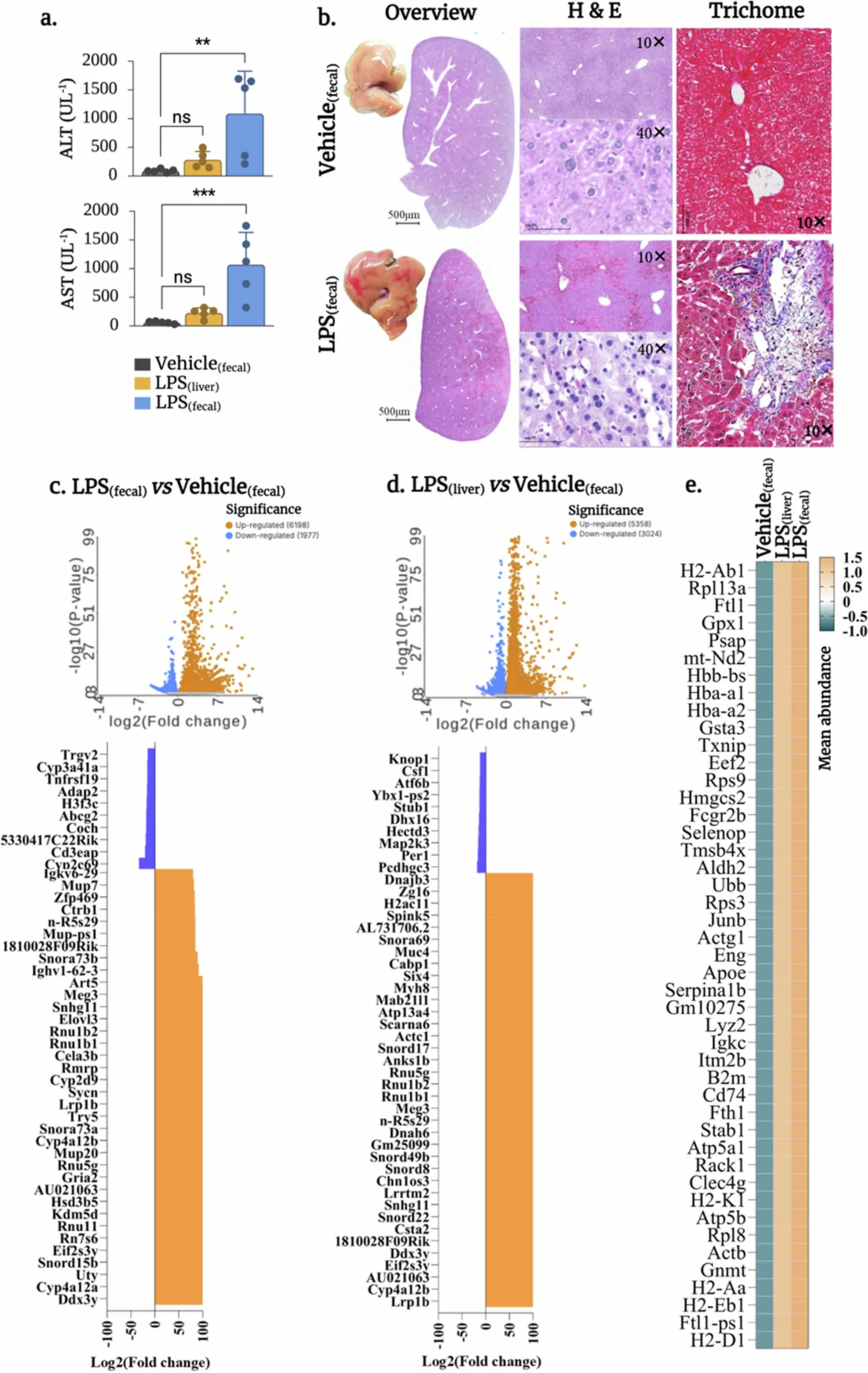
Hepatic injury and transcriptomic alterations induced by fecal- and liver-derived microbiota. The mice were transplanted with Vehicle _(fecal)_, LPS _(fecal)_, or LPS _(liver)_ microbiota as described in the Methods. The mice were examined for liver injury on day 15 post-transplantation. (a) Serum ALT and AST levels. (b) Representative gross liver morphology and large-lobe sections stained with H&E at 10× and 40× magnifications and Masson’s trichrome at 20× magnifications. (c, d) Volcano plots of significantly upregulated and downregulated genes, with representative high-fold change genes in (c) LPS _(fecal)_ vs. Vehicle_(fecal)_ and (d) LPS_(liver)_ vs. Vehicle_(fecal)_ (fold change ≥ 2; *p* ≤ 0.05). (e) Heatmap of the top differentially expressed genes (mean Z-score; *p* ≤ 0.05). Statistical significance was analyzed using a one-way ANOVA or Student’s t-test. *p < 0.05, **p < 0.01, and ***p < 0.001.

Next, we analyzed differentially expressed genes in liver tissue following microbiota transplantation. In LPS _(fecal)_ group compared with Vehicle _(fecal)_, 6,198 genes were significantly upregulated, and 1,977 genes were downregulated. Similarly, in the LPS _(liver)_ group, 5,358 genes were upregulated, and 3,024 genes were downregulated (Figure 6c,d). Among the top 50 upregulated genes, several encoded key components of antigen presentation machinery, including *H2-Ab1*, *H2-K1*, *H2-Ab*, *H2-Eb1*, and *H2-D1*, along with *B2m* and *Cd74*, indicating enhanced hepatic antigen presentation capacity. In parallel, upregulation of *Lyz2*, *Fcgr2b*, and *Igkc* suggested the activation of innate antimicrobial defenses, humoral immunity, and antibody receptor-mediated regulation, highlighting coordinated remodeling of the hepatic immune niche. Gene expression changes further implicated dysregulated immune surveillance and inflammatory responsiveness, with altered expression of *Tmsb4x*, *Junb*, *Actg1*, *Actb*, *Eng*, *Rack1*, and *Clec4g* (Figure 6e). In addition, the overexpression of *Hbb-bs*, *Hba-a1*, *Hba-a2*, *Psap*, *Txnip*, and *Selenop* was consistent with a chronic inflammatory milieu coupled to metabolic stress. KEGG pathway enrichment confirmed the activation of multiple immune-related pathways, including *TGF-β*, *TLR*, *IL-17*, and *NF-κB* signaling, alongside metabolic pathways such as fatty acid metabolism, mTOR signaling, and bile secretion. Disease-related pathways, including those associated with hepatocellular carcinoma, hepatitis, and alcoholic liver disease, were also enriched, with stronger effects in the LPS _(fecal)_ group (Supplemental Figure 8a). Together, these results demonstrated that fecal- and liver-derived microbiota from ALI mice induce hepatotoxic effects and promote liver inflammation, thereby suggesting their pathogenic role.

### Fecal- and liver-derived microbiota induced intestinal injury

Histopathological analysis of GI tract revealed that Vehicle _(fecal)_ mice displayed intact intestinal villi and abundant goblet cells, whereas LPS _(liver)_ group showed mild villus shortening and reduced goblet cell numbers. In contrast, LPS _(fecal)_ group exhibited pronounced villus atrophy, epithelial disorganization, and marked depletion of goblet cells (Figure 7a; Supplemental Figure 7b). Quantitative assessment confirmed significant reductions in villus length and goblet cell number per villus in both LPS _(liver)_ and LPS _(fecal)_ groups, with the most severe changes observed in the LPS _(fecal)_ group (Figure 7b).

**Figure 7.**
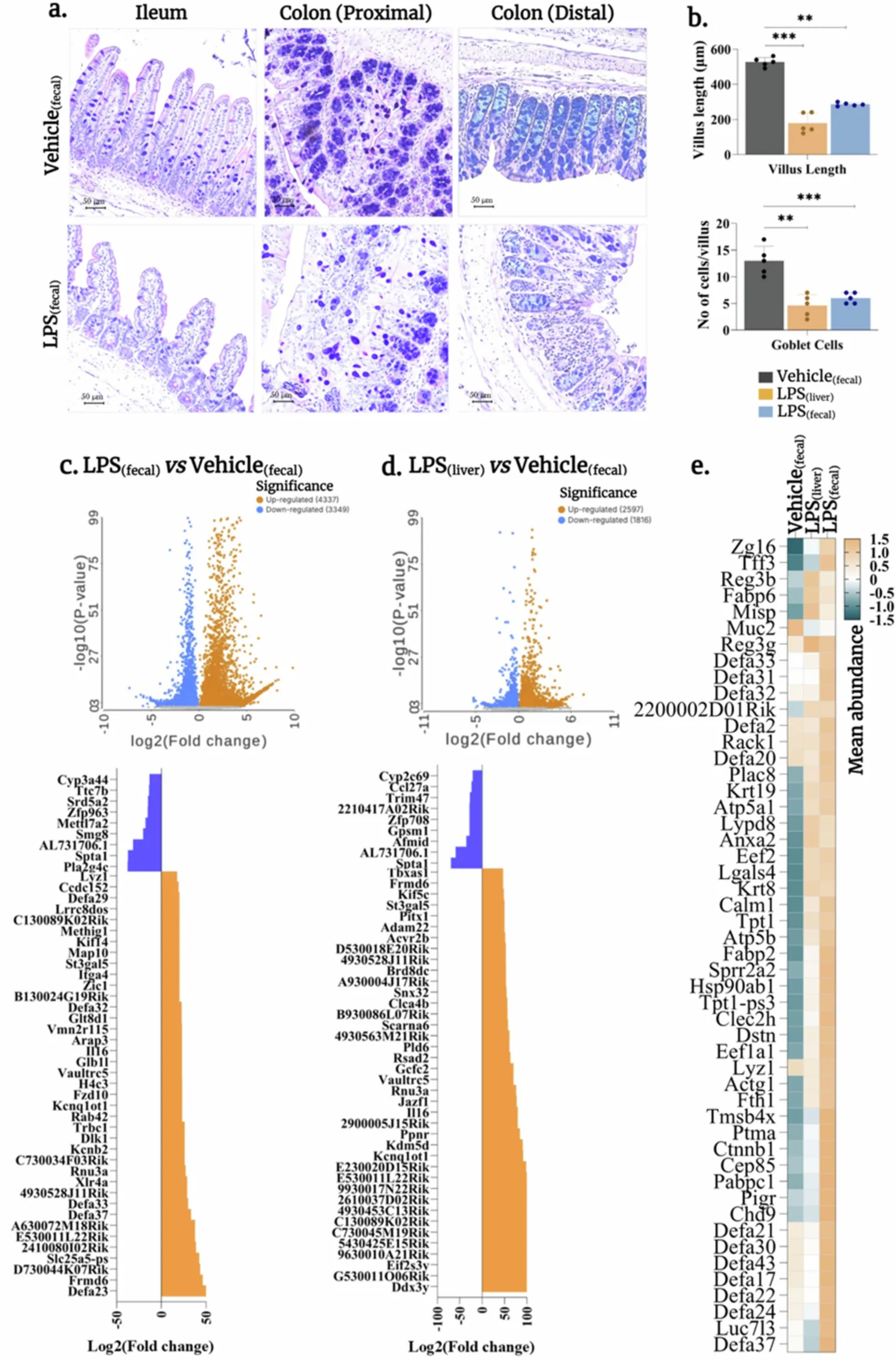
Intestinal injury and transcriptomic alterations in ECs induced by fecal- and liver-derived microbiota. The mice were transplanted with Vehicle _(fecal)_, LPS _(fecal)_, or LPS _(liver)_ microbiota as described in the Methods. The mice were examined for intestinal injury on day 15 post-transplantation. (a) Representative GI tract sections composed of ileum, proximal colon, and distal colon stained with PAS-AB at 20×  magnification. (b) Quantification of villus length and goblet cell number per villus. (c, d) Volcano plots showing significantly upregulated and downregulated genes, with representative high-fold change genes in (c) LPS _(fecal)_ vs. Vehicle_(fecal)_ and (d) LPS_(liver)_ vs. Vehicle_(fecal)_ (fold change ≥ 2; p ≤ 0.05). (e) Heatmap of the top differentially expressed genes (mean Z-score; p ≤ 0.05). Statistical significance was analyzed using a one-way ANOVA or Student’s t-test. *p < 0.05, **p < 0.01, and ***p < 0.001.

Transcriptomic profiling of intestinal epithelial cells (ECs) further demonstrated widespread alterations in gene expression, with 6,405 genes upregulated and 1,955 downregulated in LPS _(fecal)_ group, and 5,213 upregulated and 3,118 downregulated in LPS _(liver)_ group compared with Vehicle _(fecal)_ group (Figure 7c,d). A subset of the top differentially expressed genes highlighted key pathways involved in intestinal defense and barrier regulation. Members of the *Defa* (defensin) family, along with *Reg3b*, *Reg3g*, and *Lyz1*, were strongly upregulated in LPS _(fecal)_, indicating the activation of antimicrobial peptide secretion by Paneth cells and enhanced innate immune defense. In contrast, *Muc2*, the major goblet cell mucin, was reduced in both LPS _(liver)_ and LPS _(fecal)_ groups, consistent with histological evidence of goblet cell depletion and impaired mucus barrier function. Notably, *Lypd8*, which normally prevents flagellated bacteria from breaching the mucus layer, was downregulated in LPS _(liver)_ and LPS _(fecal)_ groups, suggesting impaired epithelial exclusion of motile microbes and increased risk of bacterial translocation. In parallel, altered expression of *Rack1*, a scaffold protein essential for cytoskeletal organization and immune signaling, pointed to dysregulated epithelial responses under microbial stress in LPS _(fecal)_. Additional changes included dysregulation of epithelial integrity and metabolic genes (*Fabp2*, cytochrome P450 enzymes) and altered expression of the stress-response gene *Hsp90ab1* in the LPS _(fecal)_. Collectively, these transcriptional changes reflected a shift from balanced mucosal defense toward a hyperactivated yet barrier-compromised state, potentially predisposing the intestine to increased permeability and microbial translocation in both LPS _(liver)_ and LPS _(fecal)_ groups (Figure 7e). KEGG pathway enrichment analysis revealed significant activation of immune-related pathways in LPS _(fecal)_, including *NF-κB*, Toll-like receptor (*TLR*), and *IL-17* signaling, which are central to microbial recognition and mucosal inflammation. In parallel, metabolic pathways such as fatty acid biosynthesis, cysteine and methionine metabolism, and tryptophan metabolism were also dysregulated in both LPS _(liver)_ and LPS _(fecal)_ groups, indicating metabolic reprogramming of epithelial cells under inflammatory stress. Pathways associated with disease processes, including primary immunodeficiency, necroptosis, and bacterial invasion of epithelial cells, were enriched, underscoring the pathological consequences of microbial dysbiosis and barrier breakdown (Supplemental Figure 8b).

These findings demonstrated that fecal-derived microbiota from ALI mice exert profound pathogenic effects by simultaneously disrupting intestinal barrier integrity and reprogramming hepatic inflammation, thereby amplifying gut–liver crosstalk and driving liver injury, fibrosis, and inflammation. Also, while liver-derived microbiota were less effective in mediating liver pathogenesis, the data suggested that such microbiota found in the liver during ALI may also contribute towards liver pathogenesis.

### Fecal- and liver-derived microbiota promote inflammatory immune cell activation

To investigate directly the nature of immune cells found in the livers of mice transplanted with the microbiome, we performed flow cytometric analysis. The gating strategies were described in the Supplemental Figure 9. The data revealed that microbiota transplantation induced strong immune activation both in the liver and the periphery of both LPS_(liver)_ and LPS _(fecal)_ groups, with more pronounced effects in the former group when compared to the Vehicle _(fecal)_ group (Figure 8; Supplemental Figure 10). In the liver, total leukocytes (CD45^+^) were increased by ~3-fold in LPS_(liver)_ and ~10-fold in LPS_(fecal)_ compared with Vehicle_(fecal)_. Antigen-presenting cells (MHC-II^+^CD45^+^), NK cells (NK1.1^+^CD45^+^), and myeloid populations, including monocytes (CD14^+^, CD11b^+^), Kupffer cells, macrophages, MDSCs, and M1 macrophages were markedly elevated, particularly in the LPS _(fecal)_ group (Figure 8a,b). Lymphocyte populations, including CD4^+^, CD8^+^, γδ T cells, and CD44^+^ effector T cells, were also significantly expanded (Figure 8c). Functional profiling showed increased frequencies of Th_1_ (IFNγ^+^, T-BET^+^), Th_17_ (TGFβ1^+^, IL-17A^+^), Th_2_ (GATA3^+^, IL-4^+^), and regulatory T cells (FOXP3^+^), alongside IL-2Rβ^+^ and IL-10^+^ CD4^+^ subsets in LPS _(liver)_ and LPS _(fecal)_, reflecting a mixed but pro-inflammatory skew (Figure 8d,e,f). Similarly, in the periphery, both LPS_(liver)_ and LPS_(fecal)_ transplantation significantly increased leukocytes, MHC-II^+^ APCs, NK cells, myeloid subsets (CD14^+^, CD11b^+^, macrophages, neutrophils, M-MDSCs, and G-MDSCs), and lymphocytes LPS_(liver)_ and LPS_(fecal)_ compared with Vehicle_(fecal)_ (Supplemental Figure 11a, b). Peripheral T cell subsets, including Th_1_, Th_2_, Th_17_, and Tregs, were also expanded, with particularly strong increases in Th_1_ and Th_17_ cells in LPS_(fecal)_ group (Supplemental Figure 11c, d).

**Figure 8.**
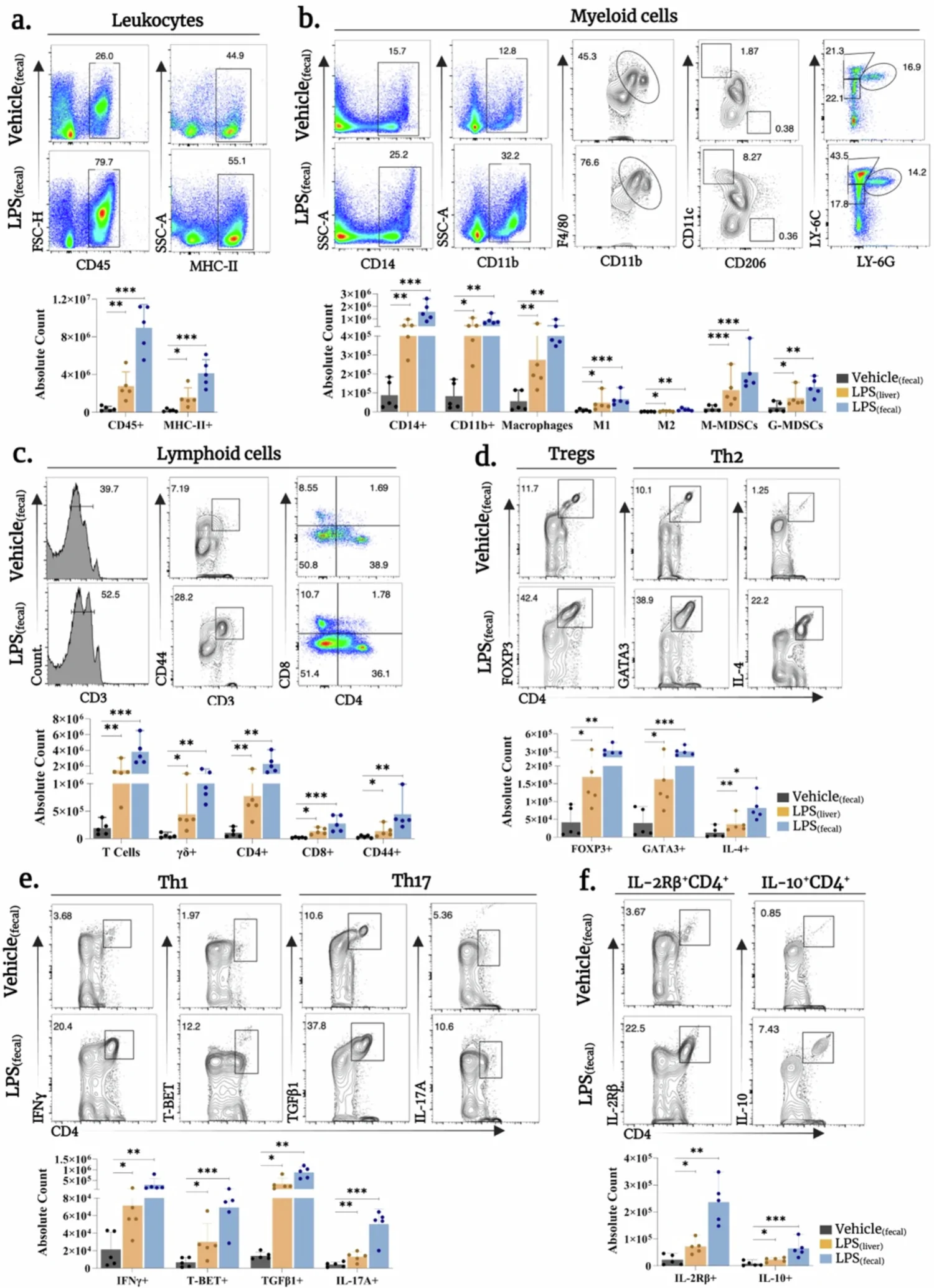
Microbiota transplantation induces hepatic immune cell infiltration and inflammatory activation. The mice were transplanted with Vehicle _(fecal)_, LPS _(fecal)_, or LPS _(liver)_ microbiota as described in the Methods. Hepatic immune cell populations were analyzed by flow cytometry on day 15 post-transplantation. Representative flow cytometric plots and quantification of (a) leukocytes; (b) myeloid cells; (c) lymphoid cells, (d) Tregs and Th_2_, (e) Th_1_ and Th_17_, and (f) IL-2Rβ^+^ and IL-10^+^ helper T cell populations. Statistical significance was analyzed using a one-way ANOVA or Student’s t-test. *p < 0.05, **p < 0.01, and ***p < 0.001.

Collectively, these data demonstrate that microbiota transplantation, especially from fecal-derived sources from ALI mice, promoted both hepatic immune cell infiltration and activation, characterized by expansion of myeloid cells and pro-inflammatory T cell subsets, thereby amplifying liver inflammation.

## Discussion

Our study demonstrates that hepatotoxins elicit distinct yet convergent patterns of hepatic and intestinal injury, underscoring the gut–liver axis as a central mediator of ALI. Among the agents tested, LPS caused the most severe phenotype, characterized by hepatocyte apoptosis, hemorrhage, and lobular disorganization, together with marked intestinal pathology, including villus blunting and mucin loss. In contrast, ConA induced transient hepatic injury, whereas SEB produced moderate but sustained damage. Across all the models, disruption of intestinal barrier integrity emerged as a defining feature, facilitating microbial translocation and systemic dissemination.

This study also provides strong evidence linking hepatotoxin-induced intestinal injury with microbial dysbiosis and translocation. The progressive increase in viable bacteria in the liver and bloodstream, particularly following LPS intoxication, indicates that intestinal barrier breakdown enables continuous microbial leakage through the gut–liver axis and portal circulation. The progressive increase in viable bacterial counts from 24 to 48 h further supports the concept of ongoing translocation rather than transient contamination.

Earlier studies have provided evidence that a compromised intestinal barrier permits pathogenic microbes and their metabolites to translocate to the liver and systemic circulation, thereby contributing to the development and progression of chronic liver diseases such as hepatitis B and C virus infections, hepatocellular carcinoma (HCC), non-alcoholic fatty liver disease (NAFLD), alcoholic liver disease (ALD), non-alcoholic and alcoholic steatohepatitis (NASH, ASH), hepatic encephalopathy (HE), autoimmune hepatitis (AIH), and cirrhosis.^49-60^ Additionally, different drug-induced liver injury (DILI) models support a time-dependent disruption of the intestinal barrier that drives microbial translocation from the gut to the liver and bloodstream. For instance, acetaminophen (APAP) overdose induces intestinal dysbiosis and facilitates bacterial migration to the liver, contributing to hepatic inflammation and injury.^61^ ^,^ ^62^ Our studies further demonstrate, by using microbiota transplantation from the gut and the liver, that these microbes are the true cause of inducing liver inflammation and injury. Notably, no prior study, to our knowledge, has directly compared fecal- versus liver-derived microbiota transfer in ALI, underscoring the novelty of our findings.

The gut microbiota is primarily composed of five dominant bacterial phyla: *Bacillota* (~79.4%), *Bacteroidota* (~16.9%), Actinomycetota (~2.5%), Pseudomonadota (~1%), and Verrucomicrobiota (~0.1%).^63^ Dysbiosis of gut microbiota is a hallmark of chronic liver diseases and is associated with a marked reduction in microbial richness. In this study, particularly in LPS-induced ALI mice, the gut microbiome exhibited a significant loss of commensal taxa, including *Butyrivibrio hungatei*, *Butyrivibrio proteoclasticus*, *Eubacterium eligens*, *Lactobacillus johnsonii*, *Roseburia intestinalis*, *Blautia hansenii*, *Lachnoclostridium* spp., and members of the family Lachnospiraceae. These taxa are major producers of short and long chain fatty acids, which serve as energy sources for intestinal epithelial cells, regulate secondary bile acid metabolism, promote anti-inflammatory immune pathways, and stimulate immunoglobulin A production.^64^ ^,^ ^65^ These alterations mirrored the microbial signatures observed in chronic liver diseases, reflecting shared dysbiotic patterns across disease etiologies.^16^ ^,^ ^66^

In patients with liver cirrhosis, gut microbial alterations are characterized by shifts in the dominant phyla *Bacteroidota* and *Bacillota*, with notable reductions in the class *Clostridia*, particularly in families *Lachnospiraceae* and *Ruminococcaceae.* Conversely, there is an enrichment of *Enterobacteriaceae* and *Pasteurellaceae* (phylum *Pseudomonadota*), along with an increase in *Streptococcaceae*.^67-69^ Patients with chronic Hepatitis B virus-related cirrhosis and hepatocellular carcinoma (HCC) exhibit reduced levels of *Lactobacillus* and *Bifidobacterium*, and an increased abundance of *Enterococcus*, accompanied by a decline in *Bacteroidota* and enrichment of *Pseudomonadota*.^70-73^ In chronic Hepatitis C virus infection, microbiota is characterized by decreased *Bifidobacterium* and increased *Prevotella* and *Faecalibacterium*.^74^ In hepatic encephalopathy (HE), *Klebsiella* species increase, and alcoholic liver disease (ALD) is associated with decreased *Clostridiales* and enrichment of pro-inflammatory *Enterobacteriaceae*.^75^ ^,^ ^76^ Patients with non-alcoholic fatty liver disease (NAFLD) and non-alcoholic steatohepatitis (NASH) show an elevated *Bacillota/Bacteroidota* ratio.^77^ ^,^ ^78^ Markedly, patients with liver cirrhosis showed distribution of *Streptococcus*, *Enterococcus, Klebsiella, Acinetobacter*, and *Pseudomonas* in bloodstream and prognosis of infections.^79^

Irrespective of gut microbiota, a small yet stable microbial population was consistently detected within the liver, possibly originating primarily from the gut. This community was dominated by *Bacteroidota* (~50%), with smaller contributions from *Bacillota* (~25%) and *Pseudomonadota* (~20%).^32^ Culture-based assays conducted in this study confirmed progressively elevated live bacterial species in the liver and blood, particularly in LPS-treated mice, consistent with active microbial trafficking through the portal circulation. Further identification showed a marked increase of community richness, such as *Staphylococcus aureus*, *Xanthomonas citri*, *Klebsiella variicola*, *Burkholderia dolosa*, *Enterococcus faecium*, *Serratia* sp., *Kitasatospora setae*, and *Prevotella melaninogenica*, along with disease progression, reflecting a pro-inflammatory microbial shift associated with intestinal barrier dysfunction and systemic immune activation, tightly linked to fibrosis progression. Notably, certain strains of *Enterococcus faecalis* are known to intensify liver damage in patients with ALD by producing a cytolytic toxin called cytolysin, underscoring the pathogenic potential of specific translocated microbes in driving hepatic injury.^80^

Beyond bacterial alterations, we identified striking virome shifts that paralleled bacteriome redistribution. Increased viral richness in the liver and blood, coupled with depletion of gut viral populations, reflects systemic dissemination following barrier disruption. Expansion of DNA phages (*Caudovirales, Siphoviridae*) and RNA viral families (*Retroviridae, Herpesviridae*), as well as species-level enrichment of *Polyomavirus*, *Mastadenovirus*, and *Herpesvirus*, aligns with recent reports linking virome instability to cirrhosis, portal hypertension, and immune activation.^81-84^ These data extend the concept that viral dysbiosis emerges early during ALI and may amplify hepatic inflammation both directly, through viral–host interactions, and indirectly, by modulating bacterial ecology.

Microbiota transplantation experiments provide direct evidence that dysbiotic microbial communities are sufficient to propagate both hepatic and intestinal pathology. Liver diseases are closely associated with immune dysregulation and inflammatory signaling. A key pathogenic mechanism underlying gut–liver translocation involves microbial metabolites such as pathogen-associated molecular patterns (PAMPs) and LPS, which engage *TLRs* on intestinal epithelial cells. Activation of these *TLR*/*MyD88*-dependent pathways induces the nuclear translocation of *NF-κB*, promoting the transcription of pro-inflammatory cytokines. These mediators, together with translocated microbial products, activate hepatic immune cells and amplify inflammatory cascades. In parallel, T cells, including resident memory, peripheral helper, and regulatory subsets, play critical roles in immune-mediated liver diseases such as autoimmune hepatitis (AIH), primary sclerosing cholangitis (PSC), primary biliary cholangitis (PBC), and metabolic-associated steatohepatitis (MASH).^16^ ^,^ ^38^ ^,^ ^42^ ^,^ ^66^ ^,^ ^85^ ^,^ ^86^

Immune profiling of recipient mice revealed enhanced hepatic immune infiltration and activation, characterized by the activation of hepatocytes, hepatic stellate cells, Kupffer cells, and recruitment of neutrophils, including M1-polarized macrophages. and pro-inflammatory T cell subsets (Th_1_ and Th_17_) following fecal or liver microbiota transplantation from ALI donors. Th_17_ cell-derived *IL-17A* plays a central role in recruiting neutrophils and activating Kupffer cells, amplifying chemokine and cytokine production.^87^ ^,^ ^88^ Concurrent overexpression of *TGF-β*, together with *IL-17A*, may promote further Th_17_ differentiation and directly stimulate hepatic stellate cells to synthesize extracellular matrix components, thereby accelerating fibrosis. *T-bet*, the master regulator of Th_1_ differentiation, enhances *IFN-γ* production, leading to macrophage activation, oxidative stress, and cytokine-mediated hepatocellular apoptosis.^89-92^ Sustained *IFN-γ* signaling may further recruit cytotoxic lymphocytes and upregulate *MHC* expression, intensifying immune-mediated liver injury. In line with these findings, transcriptomic profiling of recipient liver and intestinal epithelial cells revealed the activation of antigen presentation and inflammatory signaling pathways, including *NF-κB*, *TGF-β*, and *IL-17* cascades, following microbiota transplantation. Collectively, these results indicate that gut- and liver-derived dysbiotic microbiota can reprogram hepatic immunity, shifting the balance from immune tolerance to persistent pro-inflammatory activation, a hallmark of both experimental ALI and chronic liver disease.

In all three models of ALI, administration of hepatotoxins induced significant dysbiosis of the gut, blood, and liver microbiota, accompanied by marked intestinal and hepatic tissue injury. Furthermore, transplantation of gut- or liver-derived microbiota into microbiota-depleted recipient mice demonstrated that microbial signals exert dominant systemic effects that promote hepatic inflammation, fibrosis, and immune dysregulation. Together, these findings provide mechanistic insights into gut–liver crosstalk during ALI and support the potential of microbiome-targeted interventions as therapeutic strategies. In summary, this study demonstrates that hepatotoxin-induced liver injury is associated with gut–liver microbial dysbiosis involving both bacterial and viral components. Across three murine models of ALI, we show that disruption of intestinal barrier integrity facilitates microbial translocation to the liver via the gut–liver axis and bloodstream, resulting in hepatic immune activation and progressive liver pathology. Nevertheless, several limitations should be considered. The composition and functional characteristics of the gut microbiota differ substantially between mice and humans, and experimental murine models do not fully recapitulate the complexity, heterogeneity, and clinical course of human acute liver injury. Therefore, although our findings establish a mechanistic framework linking intestinal dysbiosis to hepatic immune dysfunction, validation in human cohorts and clinically relevant translational models will be essential to determine the applicability of these mechanisms and the therapeutic potential of microbiome-based interventions in patients.

## Methods

### Animals and in vivo models

*C57BL/6J* female mice (6–10 weeks old) were purchased from The Jackson Laboratory. The animals were housed in a sterile facility at the University of South Carolina School of Medicine and maintained on standard mouse chow and normal drinking water unless otherwise specified.

To model acute liver injury (ALI), the mice were challenged independently with ConA, SEB, or LPS following established laboratory protocols. For ConA-mediated ALI, 6-week-old mice received 12.5 mg/kg ConA (Sigma–Aldrich) intravenously in PBS.^39^ ^,^ ^40^ SEB-mediated ALI was induced in 10-week-old mice by intraperitoneal injection of dGalN (800 mg/kg; Sigma-Aldrich) combined with SEB (Toxin Technologies, FL) (50 μg/animal) in PBS (Sigma–Aldrich).^43^ ^,^ ^44^ For LPS-mediated ALI, 10-week-old mice were injected intraperitoneally with a combination of dGalN (800 mg/kg) and LPS (1 mg/kg, Sigma–Aldrich) in PBS.^48^ Vehicle-control mice received PBS intraperitoneally. For each experimental design, five animals (*n* = 5) were used per experimental group.

Survival was monitored at 12, 24, and 48  h after ConA, SEB+dGalN, or LPS+dGalN administration. At 24 and 48 h, the mice were euthanized by exposure to 5% isoflurane (Covetrus) in a closed chamber. Serum was collected to measure alanine transaminase (ALT) and aspartate transaminase (AST) activity using the ALT and AST spectrophotometric assay kits (Pointe Scientific) according to the manufacturer’s instructions. The gut content, liver, and gut tissues were collected and stored at −80 °C until further use.

### Assessment of bacterial translocation

Bacterial CFUs were quantified from liver, blood, and intestine samples collected 24 and 48  h post-challenge. The mice were sacrificed under sterile conditions in a laminar airflow hood without perfusion. Liver tissue and intestinal contents were homogenized separately in 5 mL sterile thioglycollate medium (Fisher Scientific) using a gentleMACS™ Tissue Dissociator (Miltenyi Biotech). The homogenates were passed through 100  μm strainers (EASYstrainer™, Greiner Bio-One).

Liver and intestinal homogenates were serially diluted up to 10^−3^ in sterile water and plated on Anaerobe 5% Sheep Blood Agar (Fisher Scientific). Blood samples were plated directly without dilution. The plates were incubated at 37 °C in an anaerobic chamber (Coy Vinyl) under nitrogen with 5.09% hydrogen balance. Colonies were counted after 7 d, and logCFUs were calculated. Based on microbial load, tissues and blood collected at 48 h were subsequently used for histology, metagenomics, and microbiota transplantation.^15^ ^,^ ^66^

### Histology

After 48 h of toxin challenge, the mice were euthanized by exposure to 5% isoflurane in a closed chamber and perfused through the heart with ice-cold heparinized 1× PBS (Heparin; Sigma-Aldrich). The largest liver lobe, ileum, and colon segments were harvested, flushed with PBS, and processed for Swiss rolls.^48^ ^,^ ^93^ Tissues were fixed for 24 h in 10% neutral buffered formalin, paraffin-embedded, and sectioned at 5 μm using a microtome. Replicate sections were processed by the USC Instrumentation Resource Facility for hematoxylin and eosin (H&E), Masson’s trichrome (liver), and Alcian Blue–periodic acid–Schiff (PAS-AB, intestine) staining. The sections were imaged using a Keyence brightfield microscope (VHX-7000). Histopathological evaluation was performed at 10×  and 40×  magnification. Liver sections were assessed for inflammatory cell infiltration and hepatic injury, while intestinal sections from the Vehicle, ConA, SEB+dGalN, and LPS+dGalN groups were qualitatively evaluated for inflammatory cell infiltration, epithelial integrity, villus/crypt architecture, mucin preservation, and goblet cell abundance.

### Metagenome sequencing

Microbial DNA was extracted from blood, liver, and intestinal homogenates collected 48 h post-challenge and snap-frozen in liquid nitrogen, using the AllPrep Bacterial DNA/RNA/Protein Kit (Qiagen) according to the manufacturer’s protocol. Shotgun metagenomic libraries were prepared with the Illumina DNA LP Tagmentation Kit and IDT DNA/RNA UD Indexes, producing ~300 bp fragments. Libraries were sequenced on an Illumina NextSeq 550 platform (150 bp paired-end, 300-cycle Mid Output Kit v2.5).

Quality control of raw reads, host read removal, and taxonomic annotation were performed using Kraken2 in Partek Flow (Illumina). Differential abundance analysis was performed with DESeq2. Functional metagenomic profiling was carried out using the NCBI Nephele WGSA2 pipeline.

### Liver and fecal microbiota transplantation

To deplete endogenous microbiota, 6-week-old mice received a broad-spectrum antibiotic cocktail in drinking water for 3–4 weeks, following validated depletion protocols.^32^ ^,^ ^94^ Antibiotics included kanamycin (0.5 mg/mL; Sigma-Aldrich), gentamicin (1 mg/mL; Sigma-Aldrich), colistin (1 mg/mL; Sigma-Aldrich), metronidazole (1 mg/mL; Sigma-Aldrich), and vancomycin (0.5 mg/mL; Sigma-Aldrich). In addition, mice received 100 μL of the antibiotic cocktail by oral gavage once weekly. The control mice received sterile drinking water.

For donor microbiota preparation, the donor mice were euthanized 48  h after LPS+dGalN challenge. Fresh fecal pellets were collected aseptically from GI tract immediately after euthanasia. Livers were excised under sterile conditions, rinsed briefly with sterile PBS to remove residual blood, and approximately 100 mg of liver tissue from each mouse was collected. Donor material from five mice was pooled for each transplant type. Fecal samples were homogenized directly, whereas liver tissue was mechanically homogenized in sterile PBS using a tissue homogenizer. The homogenates were diluted in sterile PBS containing 0.05% cysteine-HCl and 20% glycerol, filtered through a 100-μm cell strainer to remove large debris, aliquoted, and stored at −80 °C until use.

Following antibiotic treatment, the recipient mice were orally gavaged with 200  μL of fecal microbiota or liver microbiota suspension once daily for 3 consecutive days. The control mice received sterile PBS. Successful microbiota depletion before transplantation and bacterial recolonization after transplantation were confirmed by colony-forming unit (CFU) analysis as described in the "Assessment of bacterial translocation" section. For each experimental design, five animals (*n* = 5) were used per experimental group.

### Whole transcriptome sequencing

Two weeks post-transplantation, the mice were euthanized by exposure to 5% isoflurane in a closed chamber and perfused through the heart with ice-cold heparinized 1× PBS, and liver and intestinal tissues were collected. Intestinal epithelial cells were isolated by incubation with EDTA/DTT (Sigma–Aldrich) at 37 °C for 30  min, followed by straining and centrifugation.^95^ RNA was extracted using QIAzol (Qiagen) and the RNeasy Mini Kit (Qiagen). Pooled RNA from three mice was used to construct libraries with the NEBNext Ultra II RNA Library Prep Kit (NEB). Sequencing was performed on an Illumina NextSeq 550 platform (75 bp paired-end, High Output Kit v2.5).

Raw reads were processed for QC, aligned to the mouse genome using STAR, quantified with Partek E/M (Illumina), and analyzed for differential expression with DESeq2 in Partek Flow. KEGG pathway enrichment was performed using GSEA.

### Immune cell profiling

After 2 weeks of microbiota transplant, mice were euthanized by exposure to 5% isoflurane in a closed chamber and perfused through the heart with ice-cold heparinized 1× PBS. Liver was harvested, placed in 5 mL of Dulbecco’s modified Eagle medium (DMEM; Corning), and the liver-infiltrating mononuclear cells were isolated using the mouse Liver Dissociation Kit (Miltenyi Biotech) and gentleMACS™ Octo Dissociator (Miltenyi Biotech) with heat function program (37C_m_LIDK_1) according to the manufacturer’s protocol. Spleen samples were harvested and placed in 1× RPMI 1640 with L-glutamine (Corning) and then dissociated using Seward Stomacher 80 (BaneBio) at normal speed for 1  min. Cells were then passed through 70-mm nylon mesh cell strainers (Fisher Scientific) and centrifuged at 400× g for 7 min at 4 °C. Cells were treated with red blood cell lysis buffer (Roche) for 5 min for RBCs removal. Then, cell viability and count were analyzed using an Automated Cell Counter (TC20, Bio-Rad) after 2 times wash with fluorescence-activated cell sorting (FACS) buffer prepared with 1× PBS with 3% FBS (Sigma-Aldrich) and 1 mM EDTA (Sigma-Aldrich). Fc-receptors were blocked by incubating 1 million cells per sample with anti-mouse CD16/32 antibody (TruStain FcX, 101319, BioLegend) at a 1:100 dilution for 15 min at 4 °C. Cells were then stained for 30  min at 4 °C with a mixture of cell surface fluorochrome-conjugated antibodies. Cells were then washed twice with FACS buffer and processed for fixation and permeabilization using BD Cytofix/Cytoperm Kit (BD Biosciences) following the manufacturer’s guidelines. After washing twice with FACS, the cells were stained with an intracellular and intranuclear antibody mixture targeting cytokines and transcription factors for 30 min at 4 °C. Then, the samples were washed with FACS buffer, and the sample acquisition and spectral unmixing were performed on a spectral Flow Cytometer (BD FACSymphony A5SE), and populations were gated using the FlowJo software (v.10.8.1).^96^

The following anti-mouse antibodies were used for Flow Cytometry: CD45 (1:100) (Pacific Blue; 157211, BioLegend), CD3 (1:100) (BV510; 100233, BioLegend), CD4 (1:100) (BV421; 100543, BioLegend), CD8 (1:100) (Spark Blue 550; 100779, BioLegend), CD11b (1:100) (BUV661; 612977, BD Biosciences), F4/80 (1:100) (RY586; 753430, BD Biosciences), CD11c (1:100) (BV750; 117357, BioLegend), CD206 (1:100) (BV711; 141727, BioLegend), Ly-6G (1:100) (BUV563; 612921, BD Biosciences), Ly-6C (1:100) (BV650; 128049, BioLegend), MHC-II (1:100) (BB700; 746197, BD Biosciences), NK-1.1 (1:100) (BUV496; 569716, BD Biosciences), CD14 (1:100) (PE; 123309, BioLegend), CD44 (1:100) (BUV737; 612799, BD Biosciences), CD68 (1:100) (APC/Fire 750; 137041, BioLegend), IFN-*γ* (1:100) (Spark NIR 685; 505861, BioLegend), IL-17A (1:100) (PE/Cy7; 506921, BioLegend), TGF-β1 (1:100) (PerCP/Cy5.5; 141409, BioLegend), FOXP3 (1:100) (Alexa Fluro 647; 567462, BD Biosciences), T-bet (1:100) (PE/Fire 810; 644839, BioLegend), GATA3 (1:100) (APC; 653805, BioLegend), IL-2Rβ (1:100) (PE/Dazzle 594; 123217, BioLegend), IL-4 (1:100) (Alexa Fluro 488; 557728, BD Biosciences), and IL-10 (1:100) (BUV605; 564082, BD Biosciences).

### Statistical analysis

Each experiment included 3–7 mice per group. Statistical analyses were performed in GraphPad Prism v10.5.0 and Biorender Graph. Data are expressed as mean ± standard deviation. Comparisons between groups were made using Wilcoxon rank-sum test or Student’s t-test. Multi-group comparisons used one-way ANOVA. Statistical significance was considered when *p* < 0.05.

## Supplementary Material

R2_Supplemental file 1.docx

## Data Availability

Shotgun metagenome sequencing datasets generated during the current study are available in the SRA repository under submission ID SUB15987783 and RNA-seq datasets are available in the SRA repository under submission ID SUB15977785 in NCBI-GenBank database under BioProject ID PRJNA1416845 (http://www.ncbi.nlm.nih.gov/bioproject/1416845). This paper does not report any original code. Requests for further information and resources should be directed to and will be fulfilled by the corresponding author, Prakash Nagarkatti (prakash@mailbox.sc.edu).
