# Supplementary material for: Acute liver injury is triggered by dysbiosis and microbial translocation involving gut–liver axis
Source: Gut Microbes. 2026 Oct 19;18(1):2749977. doi: 10.1080/19490976.2026.2749977 (PMC13682391; doi:10.1080/19490976.2026.2749977)
Supplement: Supplementary file 1 [file KGMI_A_2749977_SM4060.docx]

**Supplementary figures**

**Sup Fig 1. Hepatotoxin induced ALI and gut inflammation.** Mice were injected with Vehicle, ConA, SEB+dGalN or LPS+dGalN as described in Methods. Live bacterial populations were isolated from liver, blood and gut (intestine) content were analyzed. (a) Schematic overview of the ALI models and workflow. (b) Bar graphs represent CFU count at 24 and 48 h time points. Statistical significance was analyzed using a one-way ANOVA or Student’s t-test. *p< 0.05, **p < 0.01, and ***p < 0.001.

**Sup Fig 2. Hepatotoxin induced ALI and gut inflammation.** Mice were injected with Vehicle, ConA, SEB+dGalN or LPS+dGalN as described in Methods. Mice were examined for liver injury. (a) Gross liver morphology and H&E-stained large lobe section overview of livers at 10× magnifications. (b) Detailed histopathology of those liver sections at 10× and 40× magnifications.

**Sup Fig 3. Hepatotoxin induced ALI and gut inflammation.** Mice were injected with Vehicle, ConA, SEB+dGalN or LPS+dGalN as described in Methods. Mice were examined for intestinal injury. (a) Cross-section overview (10× magnification) of the Swiss rolled GI tracts (ileum, proximal and distal colon) stained with PAS-AB. (b) Further 10× and 20× magnified views of ileum, proximal colon, and distal colon with PAS-AB along with the similar regions stained with H&E.

**Sup Fig 4. Taxonomic shifts and differential abundance of bacterial communities in liver, blood, and gut.** Mice were injected with Vehicle, ConA, SEB+d-GalN, or LPS+d-GalN as described in the Methods. Bacterial composition across taxonomic levels was analyzed by metagenome sequencing. (a-c) Volcano plots at the species level represent significant differentially abundant of bacterial species in the (a) liver, (b) blood, and (c) gut of ConA, SEB+d-GalN, or LPS+d-GalN-treated mice versus vehicle controls (fold change ≥ 1; *p* ≤ 0.05).

**Sup Fig 5. Viral diversity and taxonomic dysregulation across liver, blood, and gut during ALI.** Mice were injected with Vehicle, ConA, SEB + d-GalN, or LPS + d-GalN as described in the Methods. (a) Order-level absolute abundances, (b) Family-level absolute abundances, and (c) Viral species in liver, blood and gut were analyzed by metagenomic sequencing. Heatmap represents mean abundance (Z-score, *p* ≤ 0.05) of species level ssRNA and dsDNA viruses. Statistical significance was analyzed using a one-way ANOVA or Wilcoxon rank-sum test. *p< 0.05, **p < 0.01, ***p < 0.001, and ns=non-significant.

**Sup Fig 6. Functional profiling of microbial communities during ALI.** Mice were injected with Vehicle, ConA, SEB+d-GalN, or LPS+d-GalN as described in the Methods. Microbial functional genomics were analyzed by metagenomic sequencing. (a) Bar graph shows the abundance (in percentage; *p* ≤ 0.05) of microbial metabolic pathways. (b) Bar graph shows the abundance (in percentage; *p* ≤ 0.05) of microbial pathways related to human diseases and drug resistance.

**Sup Fig 7. Hepatic and intestinal injury induced by fecal- and liver-derived microbiota.** Mice were transplanted with Vehicle_(fecal)_, LPS_(fecal)_, or LPS_(liver)_ microbiota as described in the Methods. Mice were examined for liver and intestinal injury on day 15 post-transplantation. (a) Gross liver morphology and large-lobe sections stained with H&E at 10×, large lobe of liver sections stained with H&E and Masson’s trichrome at 20× and 40× magnifications. (b) GI tract sections composed of the ileum, proximal colon, and distal colon stained with H&E and PAS-AB at 20× magnification.

**Sup Fig 8. Transcriptomic alterations in liver and ECs induced by fecal- and liver-derived microbiota.** Mice were transplanted with Vehicle_(fecal)_, LPS_(fecal)_, or LPS_(liver)_ microbiota as described in the Methods. Mice were examined for intestinal injury on day 15 post-transplantation. Bar graphs represent immune, metabolic, and disease related pathway enrichments in LPS_(fecal)_ and LPS_(liver)_ groups compared with Vehicle_(fecal)_ in (a) liver and (b) intestine ECs.

**Sup Fig 9. Microbiota transplantation induces immune cell infiltration and inflammatory activation.** Mice were transplanted with Vehicle_(fecal)_, LPS_(fecal)_, or LPS_(liver)_ microbiota as described in the Methods. Hepatic and peripheral immune cell populations were analyzed by flow cytometry on day 15 post-transplantation. Flow cytometric plots of gating strategies of (a) liver and (b) spleen derived mononuclear cells to identify cell populations.

**Sup Fig 10. Microbiota transplantation induces hepatic immune cell infiltration and inflammatory activation.** Mice were transplanted with Vehicle_(fecal)_, LPS_(fecal)_, or LPS_(liver)_ microbiota as described in the Methods. Hepatic immune cell populations were analyzed by flow cytometry on day 15 post-transplantation. (a, b) Flow cytometric plots and quantification of leukocytes, MHC-II⁺ leukocytes, myeloid cells, macrophages, neutrophils, and lymphocytes. (c, d) Flow cytometric plots and quantification of CD4^+^ helper T cell populations. Statistical significance was analyzed using a one-way ANOVA or Student’s t-test. *p< 0.05, **p < 0.01, and ***p < 0.001.

**Sup Fig 11. Microbiota transplantation induces immune cell infiltration and inflammatory activation in periphery.** Mice were transplanted with Vehicle_(fecal)_, LPS_(fecal)_, or LPS_(liver)_ microbiota as described in the Methods. Peripheral immune cell populations were analyzed by flow cytometry on day 15 post-transplantation. (a, b) Flow cytometric plots and quantification of leukocytes, NK cells, myeloid cells, macrophages, neutrophils, and lymphocytes. (c, d) Flow cytometric plots and quantification of CD4^+^ helper T cell populations. Statistical significance was analyzed using a one-way ANOVA or Student’s t-test. *p< 0.05, **p < 0.01, and ***p < 0.001.

**Supplementary Table**

**Sup Tab 1.** Inflammatory scores for quantitative histopathological assessment ranking details of liver and GI tract at 10× and 40× magnification.

| **Score (Liver)** | **Histological findings** |
| --- | --- |
| 0 | No inflammatory infiltrate; normal architecture |
| 1 | Minimal scattered inflammatory cells |
| 2 | Mild multifocal inflammation without major tissue injury |
| 3 | Moderate inflammatory infiltrates with focal tissue damage |
| 4 | Severe diffuse inflammation with extensive tissue injury/necrosis |
| **Score (GI tract)** | **Histological findings** |
| 0 | Normal tissue; no inflammatory infiltrate |
| 1 | Mild inflammatory cell infiltration; minimal epithelial changes |
| 2 | Moderate inflammatory infiltrate with focal epithelial damage |
| 3 | Marked inflammatory infiltrate, crypt/villus distortion |
| 4 | Severe diffuse inflammation with extensive epithelial destruction, ulceration, or transmural involvement |
